# Novel linkage of health and prison data in Scotland: Investigating access to services for mental health and substance use following release from prison

**DOI:** 10.23889/ijpds.v8i2.2314

**Published:** 2023-09-14

**Authors:** Catriona Connell, Catriona Connell, Catriona Connell

**Affiliations:** 1 University of Stirling, Stirling, United Kingdom; 2 Edinburgh Napier University, Edinburgh, United Kingdom; 3 Scottish Centre for Administrative Data Research, Edinburgh, United Kingdom

## Objectives

To compare the use of NHS services for mental health and substance use (MH/SU) between people released from prison and the general population. This paper describes the data linkage and analytical process, discusses policy implications and highlights the methodological contributions for future administrative data research in public health and justice.

## Methods

Retrospective cohort study using linked Scottish health data and the Scottish Prison Service (SPS), involving all individuals released from prison in 2015 (n = 14,000), and a random general population sample (n = 70,000), matched on index date, age, sex, and postcode. Analysis will include descriptive comparison of service use between the two cohorts. Multiple regression models will be fitted to examine the influence of confounding variables in service use, and multilevel models will specifically assess cross-level geographical variation where feasible.

## Results

Results of the data linkage and analysis to date will be presented. This research will contribute to understanding the complex range of contacts people have with health services for MH/SU following imprisonment. It is the first research in Scotland to provide a national-level description of access to health services for MH/SU among people released from prison and offer a comparison to the general population. It also explores within-group differences in service access for people released from prison.

## Conclusion

The linking and analysis of multiple justice and health-related datasets will provide crucial evidence to inform future healthcare delivery for justice-experienced populations. This research also advances our understanding of public health approaches and administrative data research in justice-related contexts.

